# Effects of solar irradiance noise on a complex marine trophic web

**DOI:** 10.1038/s41598-022-16236-w

**Published:** 2022-07-16

**Authors:** Roberto Grimaudo, Paolo Lazzari, Cosimo Solidoro, Davide Valenti

**Affiliations:** 1 Dipartimento di Fisica e Chimica Emilio Segré, Universitá degli Studi di Palermo, Viale delle Scienze, Ed. 18, I-90128 Palermo, Italy; 2 National Institute of Oceanography and Applied Geophysics - OGS, via Beirut 2, I-34014 Trieste, Italy

**Keywords:** Biogeochemistry, Ocean sciences, Statistical physics, thermodynamics and nonlinear dynamics, Nonlinear dynamics, Numerical simulations, Population dynamics, Stochastic modelling

## Abstract

The analysis of experimental data of the solar irradiance, collected on the marine surface, clearly highlights the intrinsic stochasticity of such an environmental parameter. Given this result, effects of randomly fluctuating irradiance on the population dynamics of a marine ecosystem are studied on the basis of the stochastic 0-dimensional biogeochemical flux model. The noisy fluctuations of the irradiance are formally described as a multiplicative Ornstein-Uhlenbeck process, that is a self-correlated Gaussian noise. Nonmonotonic behaviours of the variance of the marine populations’ biomass are found with respect to the intensity and the autocorrelation time of the noise source, manifesting a noise-induced transition of the ecosystem to an out-of-equilibrium steady state. Moreover, evidence of noise-induced effects on the organic carbon cycling processes underlying the food web dynamics are highlighted. The reported results clearly show the profound impact the stochastic environmental variables can have on both the populations and the biogeochemistry at the basis of a marine trophic network.

## Introduction

In the political management of current scientific and social issues such as climate change and ecosystem health^1–3^, a pivotal role is played by the monitoring of the state of seas and oceans^4^. One of the most prominent aspects which characterize oceans and seas consists in their capability of absorbing atmospheric $CO_2$ and consequently mitigating global warming. Thus, the results obtained by studying the lower trophic level dynamics and marine biogeochemical processes on the basis of marine ecosystem models serves as a basic reference point.

Physical, biogeochemical, and ecological properties of marine ecosystems can be investigated through multi-nutrient and multi-plankton biogeochemical models, like BFM^5^, ERSEM^6^, PISCES^7^, ERGOM^8^, and DARWIN^9^. In our study we adopt a stochastic version of the BFM, where random fluctuating processes affecting the environmental variables are taken into account. The BFM is exploited in several fields of application, ranging from short-term forecasting^10,11^, ocean acidification^12^ and climate change^13,14^, to process studies^15–17^, biogeochemical cycling^18^ and carbon sequestration^19^.

The need of considering noise sources in the modeling of natural systems stems from their unavoidable interaction with the surrounding environment, which acts on the systems through not only deterministic but also stochastic “forces”. Random fluctuations are often responsible for the emergence of ordered phenomena from disordered dynamics. Such subtle mechanisms thus turn out to be fundamental in determining dynamic properties of chaotic systems belonging to all length scales, from microscopic physical systems like glasses^20,21^ to macroscopic ecological systems^22^. Noise can induce intriguing and counterintuitive dynamical effects in living systems such as stochastic resonance^22–26^, noise enhanced stability^27,28^, and noise delayed extinction^29^, otherwise absent in deterministic dynamics. Moreover, several examples in climate science^30^ population dynamics^31,32^, ecology^33^, epidemiology^34,35^, bioinformatics^36,37^, neuroscience and biology^38,39^, indicate that noise acts not only as a source of disorder, but also as an essential feature of the dynamics of natural systems.

The key-ingredient of these unpredictable effects is the simultaneous presence of nonlinear interactions and random fluctuations, which characterizes natural complex systems. Marine ecosystems indeed are characterized by nonlinear interactions^40^ as well as by both deterministic forcings (daily and seasonal cycles)^16,41,42^ and random fluctuations of physical variables^29,31–33,35,39,43,44^ such as temperature^45,46^. The combination of the two factors can alter the net growth rate of the phytoplankton biomass production mechanism^47^ and drive a phytoplankton system from a stability condition to another and vice-versa^48,49^.

Such examples demonstrate that, to correctly and exhaustively grasp experimental features of natural systems, the mathematical description cannot neglect the environmental random perturbations. Stochastic models can represent a powerful tool to reproduce the spatio-temporal evolution of marine ecosystems in a better agreement with field data, shedding a new light on the hidden mechanisms which govern the dynamics of natural ecosystems, suggesting strategies and indicating possible solutions about urgent climatic issues. Stochastic models for the climate, for example, provided a means of proving that human emissions of carbon dioxide cause the increase of atmospheric temperature^50–52^. In this sense, the separation of the deterministic signal (the large-scale dynamics of the climate) from the noise (the short-range changes in the weather) has been at the basis of methods developed for identifying particular features of human-induced effects in the analysed experimental data^53^.

As for biogeochemical and ecological models, until now the attention has been mainly focused on the effects stemming from random temperature fluctuations^54–56^. However, in ecological systems also light plays a fundamental role in driving their dynamics. To the best of our knowledge, what is reported in this paper is the first attempt to analyse the dynamic aspects related to the randomicity of the solar irradiance. This idea finds its root in the examination of experimental time series of the irradiance parameter which account for the intrinsic stochastic character of light.

In the present work, basing on previous studies^43,57–60^, the stochastic character of light is taken into account by modeling the solar irradiance dynamics as a Brownian particle subject to a multiplicative self-correlated Gaussian noise. The zero-dimensional stochastic biogeochemical flux model (SBFM)^54^, then, simulates a planktonic food web made of nine populations under the influence of a randomly fluctuating irradiance. Moreover, it accounts for biogeochemical cycles of carbon, phosphorus, nitrogen and silicate as well as the evolution of dissolved and particulate organic matter.

The paper aims at showing that irradiance parameter does have an intrinsic stochastic component, and that such a stochasticity has a significant impact on the dynamics of plankton community and the resulting structure. Our results demonstrate that the coefficient of variation (the ratio of the standard deviation to the mean value over the ensemble of realizations) in the stochastic process reaches a maximum in correspondence to critical values of  the random fluctuations' intensity for all the bio-variables. Irradiance random fluctuations mainly affect bio-physical processes related to plankton dynamics (e.g., photosynthesis) but such ‘localized’ noisy perturbations on the lowest trophic levels can be reverberated, and indirectly affect the highest levels too. The analysis of the probability density functions reveals the ergodic character of the network dynamics, as well as the noise-induced generation of an out-of-equilibrium steady state, i.e. of a dissipative state sustained by the existence of a continuous series of perturbations, filtered by the nonlinear dynamics describing the plankton response to light intensity. This last aspect particularly shows how the interplay between random and deterministic fluctuations of the environmental parameters is fundamental in determining the basic dynamic features of complex systems like a marine ecosystem.

## Results

This section is devoted to show results and to highlight eventual effects of the interplay between the nonlinearity characterizing the system dynamics and the presence of noisy fluctuations for the irradiance variable.

### Analysis of experimental data

**Figure 1:**
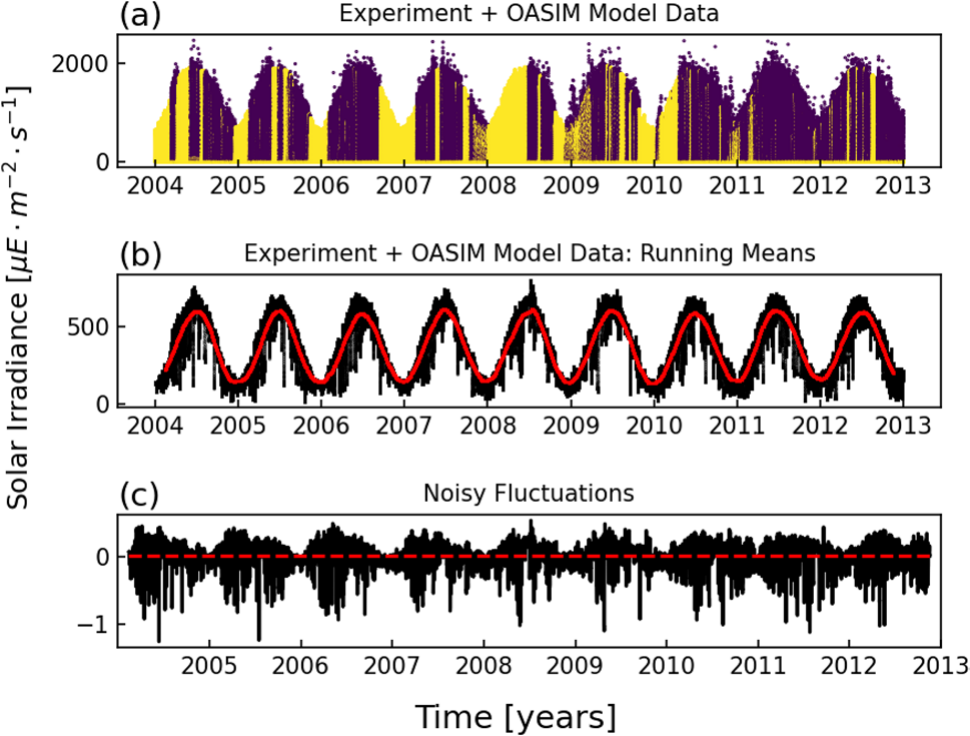
(**a**) Experimental data (purple points) of the stochastic solar irradiance collected by the Boussole buoy in a time-window of 9 years (2004-2013); the yellow points are the data generated by the OASIM model used to fill the gaps present in the experimental time-series due to malfunctioning of the buoy. (**b**) Daily (black points) and three-month (red points) running mean of the light curve in panel (**a**). (**c**) Irradiance noisy fluctuations (INF), obtained by subtracting the three-month running mean curve (3MRM) from the daily running mean one (DRM) and normalizing with respect to the mean value of 3MRM ($\overline{3MRM}$), namely $INF = (DRM - 3MRM) / \overline{3MRM}$; the red line represents the mean value of such fluctuations. Data already presented and validated in^61^.

The need of taking into account noisy fluctuations of such an environmental variable is well demonstrated in Fig. 1. In the first panel (a) the experimental time behaviour of the irradiance is shown. This noisy curve is based on the experimental data (purple points) of the Boussole buoy located in the Gulf of Lion, collected over a period of nine years, precisely from 2004 to 2013. The time series of the experimental data presents quite a few gaps in time due to the malfunction of the buoy. This aspect has been remedied by merging the experimental data with those of the OASIM model validated for the Boussole site^61^ (yellow points). The latter is a multispectral atmospheric radiative transfer model that is in turn forced by experimental-model data based on ECMWF ERAINTERIM reanalyses which provide, for example, cloud cover data. The radiative model is partly stochastic since it considers the effects stemming from the presence of clouds, averaged along a single day (this explains why the yellow points are slightly less scattered). We see that the OASIM model accurately reproduces the profile which emerges from the experimental data. Further, we stress that the experimental data are only used in this initial analysis. In the biogeochemical simulations the irradiance signal is fully reconstructed starting from a realistic seasonal cycle combined with a range of different random fluctuations, and the information from OASIM is not used. In the second panel (b) the daily (black points) as well as the three-month (red points) running mean of the experimental series are plotted. Figure 1c shows the irradiance noisy fluctuations (INF) which have been obtained by subtracting the three-month running mean curve (3MRM, red curve in Fig. 1b) from the daily running mean one (DRM, black curve in Fig. 1b) and normalizing with respect to the mean of the 3MRM ($\overline{3MRM}$), namely $INF = (DRM - 3MRM) / \overline{3MRM}$. We see that a seasonal overall trend with higher oscillations during the winter time can be seen, implying that the characteristics of the noise may change over the year. Moreover, a slight imbalance between positive and negative values of the noisy fluctuations (that is, different values of the maximum fluctuation intensity) is present. The physical reason for the occurrence of such an aspect can be ascribed to the fact that the maximum value of solar irradiance corresponds to that measured during a sunny day. Conversely, the minimum level tends to zero corresponding to a dense darkness. While the former is close to the mean value of the solar irradiance (most of all in summer), the latter is much further away and then a natural asymmetry arises in the random fluctuations. However, it should be noted that, apart from the intense spikes, the asymmetry is not so pronounced, as proved by the mean value (red line in Fig. 1c) which is practically zero, namely $0.4\%$ of the $\overline{3MRM}$. Therefore, basing on this last observation, to model the noise affecting the irradiance dynamics, as a first approximation we consider a symmetric Gaussian autocorrelated noise as described in the next subsection.

On the basis of such experimental results, we postulate the hypothesis that random fluctuations of light cannot be neglected, most of all in the study of ecological systems where light profoundly determines the system dynamics, governing fundamental processes at the basis of of the food web.

### Solar irradiance

**Figure 2:**
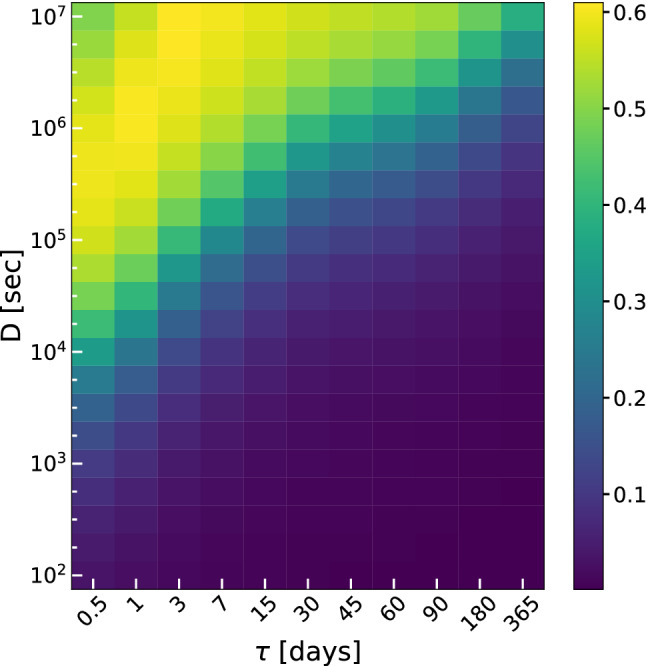
Coefficient of variation ($CV=\sigma / \mu$) of irradiance resulting from numerical integration of model equations for 231 $D-\tau$ different scenarios.

The solar irradiance forcing is derived considering a deterministic seasonal oscillation combined with an Ornstein-Uhlenbeck process. The coefficient of variation (CV) of simulated light forcing, Fig. 2, $CV=\sigma / \mu$ ($\mu$ and $\sigma$ being mean value and standard deviation calculated over both time and numerical realizations), is shown for 231 $D-\tau$ pairs. *D* and $\tau$ represent the intensity of a Gaussian noise source and the auto-correlation time of the fluctuations, respectively (see Eqs. (2) and (3)).

Each pixel represents the mean value on time of CV calculated with respect to 1000 different stochastic realizations.

It is easy to see the agreement between the results obtained from the numerical integration and the theoretical ones derivable from Eq. (5) by putting $\text {var}\{F_L(0)\}=0$ and $t \gg 1$, getting $\sigma ^2_L=D / 2\tau$. In Fig. 2, indeed, the maximum values of $\sigma$ lie in the upper left part of the plot corresponding to small (high) values of $\tau$ (*D*). As it is clear the values of *D* have been chosen in order to obtain a relative standard deviation ranging from $5\%\mu$ to $60\%\mu$. We underline that, in this case, it is possible to interchangeably consider $\sigma$ and CV since the dependence of CV on *D* and $\tau$ does not differ from that of $\sigma$ (meaning that the dependence of $\sigma$ is not altered by dividing by $\mu$) (results not shown).

### Effects on population dynamics

**Figure 3:**
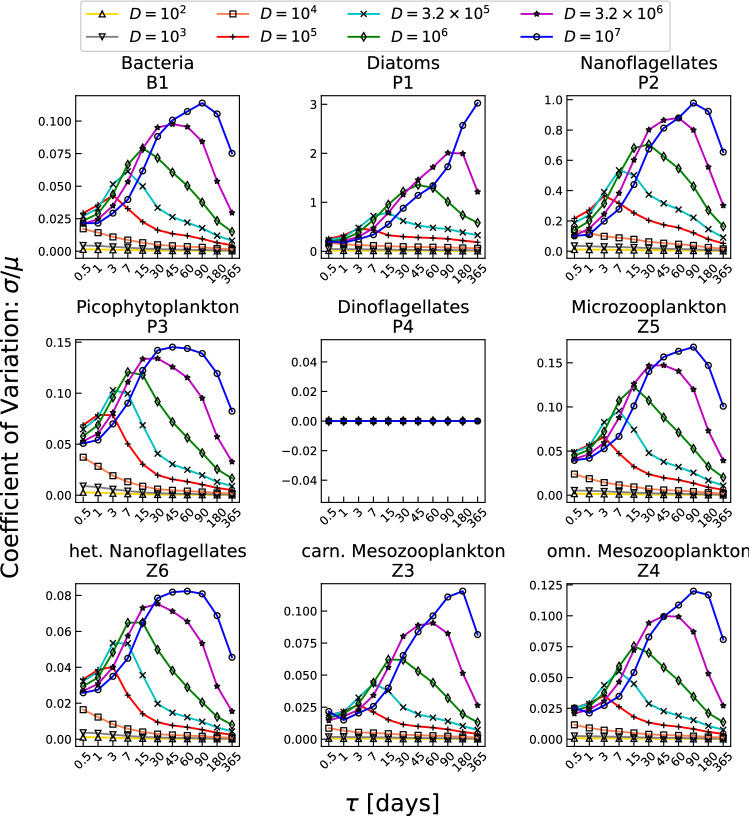
Coefficient of variation ($CV=\sigma / \mu$) of the nine planktonic populations resulting from numerical integration of model equations plotted versus the considered values of $\tau$; the different curves are related to different values of the noise intensity *D*.

In this section the noise-induced effects on the population dynamics are examined. The nine planktonic populations present a different qualitative behaviour of the CV, compared to that of the irradiance. In this case, the CV is characterized by a strong non-monotonic dependence on the parameter $\tau$. This aspect can be appreciated in Fig. 3 where different curves of CV *versus* the time correlation parameter are shown for different fixed values of *D*.

The existence of a maximum value for CV can be appreciated for each species. Although the qualitative behaviour is the same for all strains, particular attention has to be payed on diatoms and nanoflagellates. All the other species, indeed, present a percent variation of standard deviation between $2\%$ and $15\%$. In the case of nanoflagellates, instead, the *D*-dependent range is $20-90\%$, while diatoms reach values over the $100\%$ for the highest values of *D*. Therefore, these two species, in particular, and the whole system, in general, are extremely sensitive to the auto-correlation time which characterizes the noise.

We note that the different curves related to the different selected values of *D* approach the horizontal axis, tending asymptotically to vanish as $\tau$ increases. Such a behaviour can be explained by the fact that high values of $\tau$ give rise to a more correlated dynamics, so that $\tau \rightarrow \infty$ implies fully correlated time-behaviours corresponding to the deterministic case. In this instance, then, all the different realizations give the same results, making the standard deviation vanish. The same happens, independently of the value of $\tau$, for low values of noise intensity for which the corresponding curves approach the same almost vanishing value (see orange, gray and yellow lines). Differently from the previous case, when $\tau \rightarrow 0$ the noise tends to a delta-correlated noise, that is a white noise; for $\tau \ne 0$, instead, the noise spectrum is not flat, being characterized by a Cauchy-Lorentz distribution. The strong nonmonotonicity of CV with respect to $\tau$, emerging when there are relatively high values of CV, implies a greater variability of the system biomass. Lower values of CV indicate that the system dynamics is less influenced by the presence of noise where very little or no differences with respect to the deterministic case are present. Conversely, high values of CV clearly demonstrate the remarkable signature of the presence of an impacting noise source. It is interesting to note that the noise influence on the ecosystem strongly depends on both $\tau$ and *D*, that is, just an intense noise is not enough to generate a greater response of the ecosystem. In particular, experimental data are characterized by a CV approximately equal to 0.3^61^, which corresponds to values of *D* and $\tau$ lying on the diagonal strip in Fig. 2 ranging from $(\tau ,D)=(0.5,10^4)$ to $(\tau ,D)=(365,10^7)$. Finally we note the presence of a noise suppression effect. High values of *D*, indeed, can generate slight effects when the correlation time $\tau$ does not take on suitable values.

The results shown here are an extension of the previous work by Benincà et al.^56^. There, the authors analyse a simpler, less realistic model of two interacting populations, whose dynamics is affected by a randomly fluctuating temperature. In that case, moreover, the deterministic oscillations of the temperature are suppressed, and the system exhibits intrinsic Lotka-Volterra oscillations whose frequency match with the characteristic one(s) of the noise. On the contrary, here, the observed maximum response (see Fig. 3) cannot be interpreted as a synchronization effect, since our model does not present intrinsic Lotka-Volterra-like oscillations and the periodic population variability is only due to the deterministic forcing(s).

The nonmonotonic behaviour of the CV can be then interpreted as the signature of the intimate interplay between the ecological system and the noise. This interplay, indeed, has a pivotal role in both determining the dynamics of the populations and defining the characteristics of the ecosystem.

In Fig. 3 it can be observed that the value of $\tau$ for which CV is maximum strongly depends on the noise intensity *D*. In particular, it is possible to note that the peaks in Fig. 3 move towards higher values of $\tau$ as the noise intensity increases. Thus, Fig. 3 demonstrates that the maximum-response effect to the random fluctuations is sensitive to the noise intensity *D*.

However, it is important to underline that the response of the system to the noisy signal does not depend on the yearly oscillations induced by the deterministic forcings. Indeed, by considering constant the deterministic part of all external forcings (temperature, irradiance, wind and salinity), the non monotonic behaviour of CV with respect to both $\tau$ and *D* is still present, provided that the populations are not extinct (plot not shown). In this scenario indeed, besides dinoflagellates, diatoms and nanoflagellates are practically extinct as well, exhibiting thus a constant vanishing variance. All the other strains, instead, present qualitatively the same nonmonotonicity with only slight differences (shift of the peaks and different mean values of the CV curves), probably due to the extinction of diatoms and nanoflagellates which causes relevant differences in the system dynamics. More specifically, the system’s response seems to depend on both the noise intensity and the correlation time (see Fig. 3).

**Figure 4:**
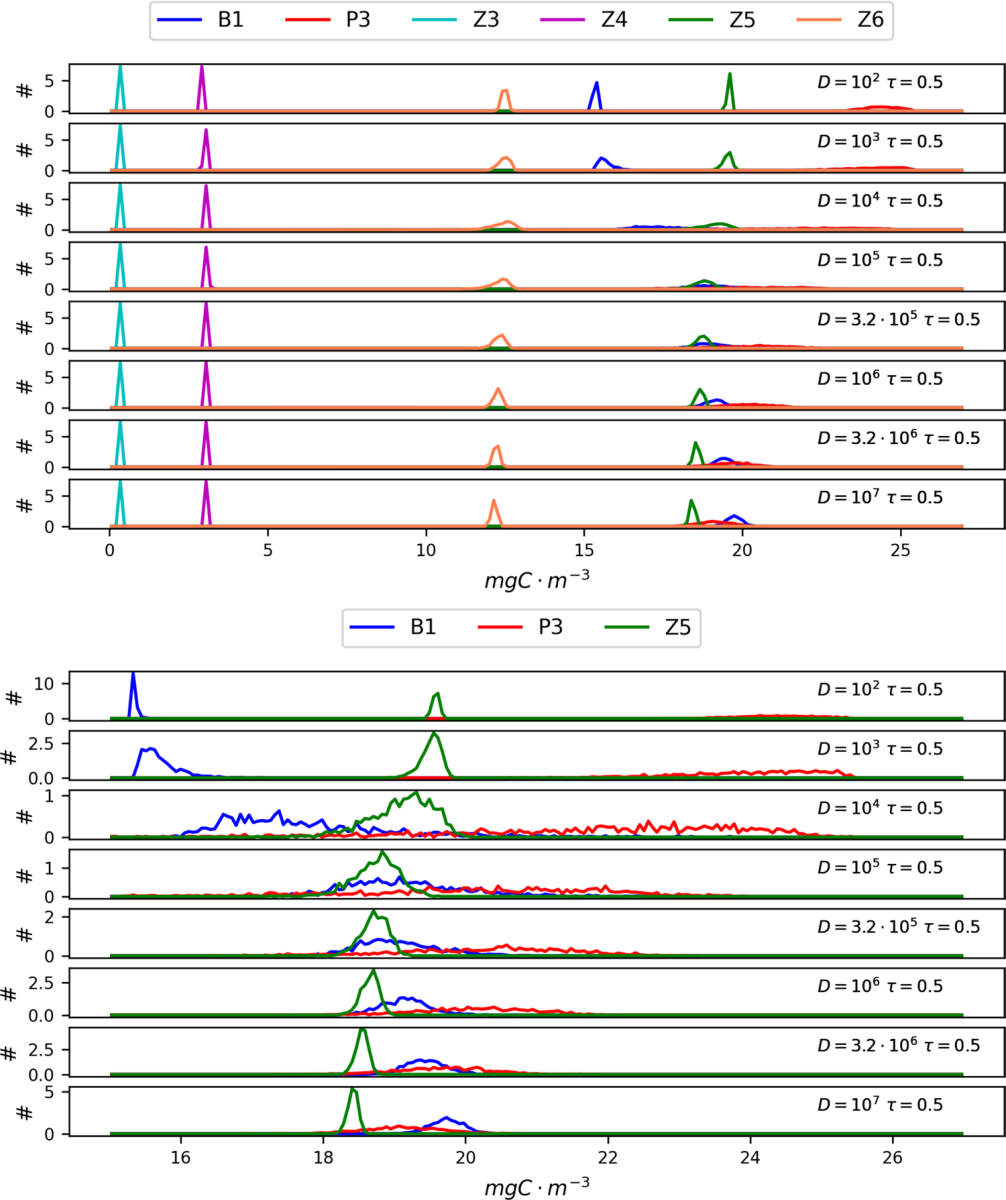
Dependence of the probability density functions of non-vanishing populations on the parameter *D* for $\tau =0.5$. The curves are normalized within the interval taken into account. For this reason the relative peaks of the curves in the bottom panels have different values compared to those of the top panels. However, the figure aims at showing the existence of the value of the noise intensity for which the system is more sensitive as well as the generation of a stationary out-of-equilibrium state induced by the noise.

In this scenario (absence of seasonal driving) we have studied the dependence on both parameters *D* and $\tau$ of the probability density functions (PDFs) of the non-vanishing populations. In Fig. 4, the PDFs of bacteria (B1), picophytoplankton (P3), microzooplankton (Z5) and etherotrophic nanoflagellates (Z6) are plotted for $\tau =0.5$ and eight different values of the parameter *D*.

We see that the mean value and the variance of these populations are strongly affected by the presence of random fluctuations in the irradiance. Specifically, as the noise intensity increases the mean values of picophytoplankton and bacteria concentrations exhibit a shift. In particular, the results indicate that picophytoplankton is disavantaged by the presence of a noisy component in the irradiance, which indeed tends to inhibit its ability to absorbe the solar light, slowing down its growth. As a consequence, since phytoplankton and bacteria compete for the same resources, as the former declines the latter are favoured, with a compensation mechanism which allows their predators (zooplankton populations) to be almost not affected by the noisy behaviour of the irradiance. Further, we note that for intermediate values of the noise intensity ($D = 10^4 - 10^5$) a maximum of the variance occurs (the PDFs are clearly spread on a wider range of values). Such an effect indicates that the noisy behaviour of irradiance strongly influences the whole ecosystem dynamics. Moreover, the nonmonotonic behaviour of the variance (its PDFs become larger and then tighter again as the noise intensity increases) indicates that the noise pushes the ecosystem away from equilibrium, driving it towards a non-equilibrium steady state. Finally, we note that the nonmonotonic behaviour of CV as a function of the noise intensity remains also in the presence of seasonal driving.

**Figure 5:**
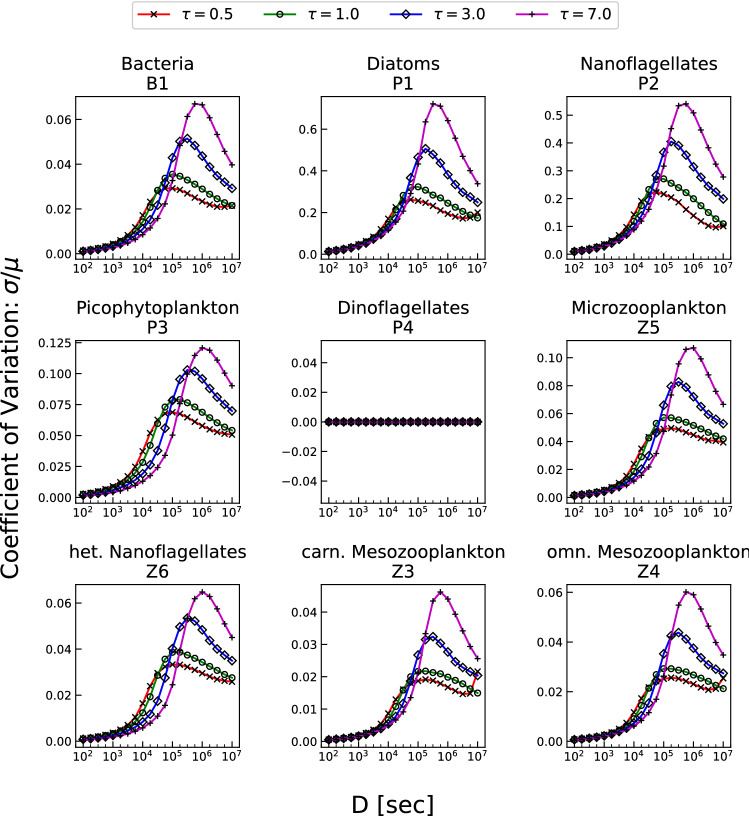
Coefficient of variation ($CV=\sigma / \mu$) of nine planktonic populations resulting from numerical integration of model equations plotted versus the considered values of *D*; different curves correspond to different values of the correlation time $\tau$.

Figure 5 shows indeed the nonmonotonic response of the ecosystem to the change of *D* when the deterministic seasonal cycling of the four environmental parameters (temperature, irradiance, wind and salinity) is present. It is easy to observe that also in this instance the major noise-induced effect appears in nanoflagellates and diatoms with a percent standard deviation of 50$\%$ and 100$\%$, respectively. The coalescence of different curves (related to different values of $\tau$), as *D* decreases, is due to the fact that for $D \rightarrow 0$ the impact of the noise is negligible and the evolution of the system practically resembles the deterministic one. On the contrary, for higher values of *D* remarkable differences arise and clear peaks of CV appear in the considered range of variation.

These plots show that, for a fixed value of $\tau$, there exists a value of the noise intensity for which the planktonic concentrations are maximally spread around their mean values (corresponding to the maximum value of CV and then of the variance). Moreover, such a nonmonotonic behaviour suggests the presence of a resonance, which can be interpreted as the effect of the interplay between the nonlinearity of the system and the environmental random fluctuations.

Also in this case, the interplay between the two parameters *D* and $\tau$ in determining and characterizing the dynamics of the ecosystem transparently emerges. The value of *D* corresponding to the maximum value of CV, indeed, basically depends on the specific value of $\tau$.

Finally, we point out that the different dynamic scenarios identified by the *D*-$\tau$ couples can be experienced by the system during the year, since the two parameters may seasonally vary depending on the different weather conditions. In other words, a seasonally varying noise (see Fig. 1c) may cause the nine populations explore different regions of the *D*-$\tau$ space during the year. Therefore, the results reported in this paper can highlight the detectable yearly variability of a marine ecosystem which does not stem from the deterministic seasonal variation of environmental parameters.

### Effects on the organic carbon

**Figure 6:**
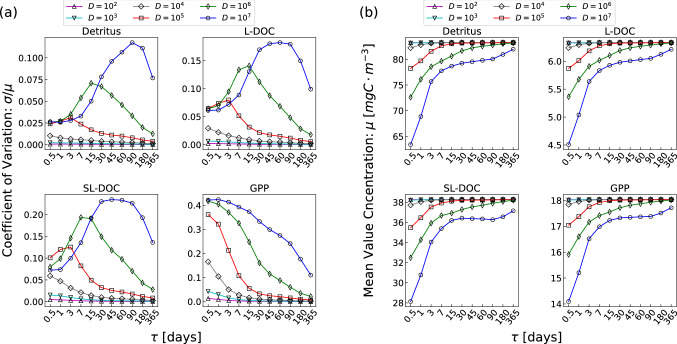
(**a**) Coefficient of variation ($CV=\sigma / \mu$) and (**b**) mean value concentration ($\mu$) of detritus, labile dissolved organic carbon (L-DOC), semi-labile dissolved organic carbon (SL-DOC) and gross primary production (GPP) resulting from numerical integration of model equations plotted versus the considered values of $\tau$; the different curves are related to different values of the correlation time *D*.

In this subsection the effects of the irradiance noise on the biogechemistry are analysed. In Fig. 6 the dependence on $\tau$ of both the CV [panel (a)] and the mean value concentration [panel (b)] of detritus, labile dissolved organic carbon (L-DOC), semi-labile dissolved organic carbon (SL-DOC) and gross primary production (GPP) are shown. All these biogeochemical properties are correlated with carbon cycling. Gross primary production is related to the amount of carbon entering in the ecosystem, and is related to the maximum energy available in the ecosystem progressively dissipated in the trophic web. Gross primary production is directly affected by light fluctuation and its CV shape is very similar to that of the irradiance, Fig. 2. We selected also detritus and DOC because they are important indicators for the carbon cycling dynamics and are related to the cycling of chemicals like heavy metals^62^. The different curves, related to different values of *D*, approach the same (vanishing) value for large $\tau$. As previously discussed for the CV [Fig. 6(a)] of biomass concentrations, this circumstance is due to the fact that, in this case, the system dynamics tends to the deterministic case, characterized by a unique possible realization implying a vanishing standard deviation. For high correlation times thus the system is insensitive to the noise intensity. On the contrary, for small values of $\tau$, different values of *D* lead to significant differences of the variance. In particular, detritus, L-DOC and SL-DOC exhibit a clear non-monotonic behaviour whose maximum value depends on the combined values of *D*-$\tau$. Only the GPP presents a decreasing monotonic behaviour.

The dependence of the mean value concentration on $\tau$, instead, is qualitatively the same for all the four parameters. Also in this case we can note a diversification with respect to *D* occurring at small $\tau$ and a (deterministic) constant value arising for low (high) values of *D* ($\tau$).

These results manifest that not only the population dynamics, but also all the biogeochemical processes are profoundly affected by the presence of stochastic environmental variables. The values and the behaviour of the examined quantities are indeed determined by the intimate interplay between the intensity and the time correlation of the noise fluctuations.

## Concluding discussion

One of the most important prerequisites for an appropriate assessment and management of risks associated with the climatic variations is to understand the processes that regulate the chemistry of the elements in seawater. These processes, indeed, are connected with potential risks for marine ecosystems both as a function of variations of chemical and physical conditions of the marine environment and in terms of specific bioavailability and possible biomagnification along the trophic web. Variations of basic processes can cause a series of macroscopic effects on all the components of the earth system, affecting more and more our planet. Crucial modifications in the main biogeochemical cycles and major photosynthetic processes, for example, could be induced by such changes during the next decades, with dramatic consequences on the functioning of marine ecosystems.

The mathematical models used to foresee the impact of the changes in the basic biogeochemical processes on the trophic network cannot neglect the intrinsic stochasticity characterizing the environmental parameters. The consideration of stochastic processes, indeed, allows to catch phenomena otherwise neglected: the noise, for example, can have a profound impact on the steady-state of an aquatic ecosystem. In phytoplankton population dynamics, changes of limiting factors such as the light intensity and nutrient concentration cause a passage from a stability condition to another and vice-versa^48,49^. Other physical variables, such as temperature, can further modify the spatio-temporal behaviour of the net growth rate of the phytoplankton biomass production mechanism^47^.

With regard to this aspect, the present work is a first step aiming at proposing an integrated experimental-modelling approach. The model we used is the Biogeochemical Flux Model (BFM) which simulates the dynamics of a marine ecosystem composed of nine populations, considering several biological and chemical processes at the base of such a network system, like respiration and excretion *etc.*. This is the first attempt of investigating the effects of the random fluctuations of solar irradiance on the dynamics of a natural complex system. The solar irradiance fluctuations affect the dynamics of the ecosystem since sunlight governs several fundamental processes like the photosynthesis. The use of the stochastic version of the BFM brings to light the effects of random fluctuations on the trophic web and shows the role played by the environmental noise on the ecosystem dynamics.

By examining the nine-year temporal series of the irradiance, collected by the Boussole buoy on the Gulf of Lion, we extracted information about the main features which characterize the random fluctuations of the light intensity. On this basis, we modeled the noise affecting the irradiance with a zero-mean multiplicative Ornstein-Uhlenbeck process, imposing the limited range $[-100\%,100\%]$ for the fluctuations as suggested by the results of our analysis.

The two parameters of such a stochastic process are the intensity of the noisy fluctuations (*D*) and their correlation time ($\tau$). We explored 231 scenarios identified by different (fixed) values of *D*-$\tau$ couples performing, for each scenario, sets of 1000 realizations, and studying the emerging effects on the population dynamics. In this way we generated *D*-$\tau$ maps where our system ideally moves drawing trajectories during the year which depend on to the seasonal changes of the two noise parameters (*D* and $\tau$) clearly highlighted by the experimental data for the irradiance fluctuations. In particular, we studied the dependence of the coefficient of variation (CV) of the biomass concentrations of the nine strains on both *D* and $\tau$. We found a relevant nonmonotonic behaviour of CV *versus* the two noise parameters. Such a nonmonotonic behaviour indicates the existence of a region in the parameter space where the ecosystem is highly sensitive to the random fluctuations, exhibiting a maximum response to the noisy component of the irradiance^56^.

In light of these results, we wish to note that our analysis contributes to reveal the presence of a non-trivial noise-induced dynamics, where the ecosystem is pushed away from the deterministic attractor and is more realistically driven towards a new non-equilibrium steady state. This noise-induced state disappears after removing the noise source and the system goes back to the deterministic equilibrium state. This confirms that the noise induces no transition towards a new equilibrium point, simply maintaining the ecosystem in an out-of-equilibrium stationary regime.

Finally, a clear nonmonotonic dependence on both the parameters $\tau$ and *D* has been observed for the CV of detritus, labile dissolved organic carbon and semi-labile dissolved organic carbon, which proves that also the biogeochemistry is deeply influenced by the environmental stochasticity.

Therefore, our results demonstrate that the variance of the stochastic irradiance (determined by the values of *D* and $\tau$) indirectly generates variability in the population concentrations and that the maximum variance on the biomass does not correspond to the maximum of variability of the light noise. Such a nonmonotonicity unveils the remarkable interplay between the noise and the nonlinearity which governs the system dynamics.

## Methods

### The biogeochemical flux model

**Figure 7:**
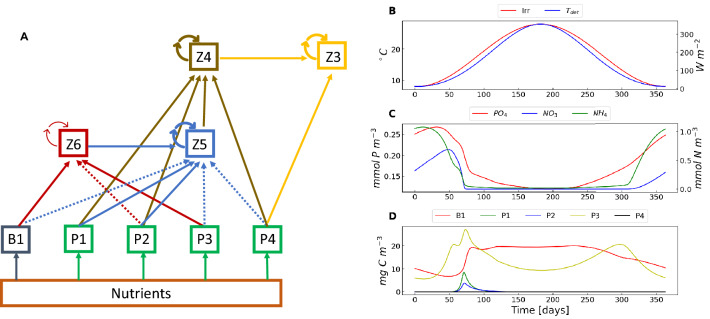
(**A**) Trophic web interactions described by the BFM. The considered nine plankton populations are: carnivorous mesozooplankton (*Z*3), omnivorous mesozooplankton (*Z*4), microzooplankton (*Z*5), heterotrophic nanoflagellates (*Z*6), bacteria (*B*1), diatoms (*P*1), nanoflagellates (*P*2), picophytoplankton (*P*3), and dinoflagellates (*P*4). An arrow directed from one box to another indicates a predation flux. Solid arrows denote a higher preference for a specific prey while dashed ones indicate a lower preference. A looping arrow on the box itself denotes cannibalism. Deterministic time behaviour during the tenth (last) year of simulation of: (**B**) temperature ($T_{det}$, $^\circ$ (**C**) and irradiance (*Irr*, $W~m^{-2}$); C) inorganic nutrients phosphates ($PO_4$, $mmol~P~m^{-3}$), nitrates ($NO_3$, $mmol~N~m^{-3}$), and ammonia ($NH_4$, $mmol~N~m^{-3}$); (**D**) bacteria, diatoms, nanoflagellates, picophytoplankton, and dinoflagellates, all expressed in $mg~C~m^{-3}$.

The deterministic biomass-based BFM includes a conspicuous number of studies and applications, namely^10–19^, to cite a few. The deterministic configuration of the BFM has been developed to accurately reproduce the dynamics of the following plankton functional types (PFTs): primary producers (phytoplankton), predators (zooplankton), and decomposers (bacteria). Within these functional classes more specific functional subgroups can be identified to better define the planktonic food web. Diatoms (P1), flagellates (P2), picophytoplankton (P3) and dinoflagellates (P4) constitute the class of primary producers. The class of predators consists of: i) microzooplankton, composed of heterotrophic nanoflagellates (Z6) and microzooplankton (Z5); ii) Mesozooplankton, distinguishable in omnivorous (Z4) and carnivorous (Z3). Bacteria (B1) are responsible for the basic aspect of recycling organic compounds in inorganic constituents such as nitrates, phosphates and silicates. The BFM provides a much more realistic description of the microbial food-web trophic interactions than the remarkably simpler Lotka-Volterra type models^63–65^. It accounts, indeed, also for the major biogeochemical processes that form the basis of the dynamics of a pelagic marine ecosystem. The BFM simulates, for example, the cycles of nitrogen, phosphorus, silica, carbon, and oxygen in water due to plankton activity. A sketch of the trophic network is shown in Fig. 7.

The state of each plankton functional group is formalised as a vector whose components are given by the primary constituent concentrations. The physiological condition of bacteria, for example, is defined by the set of intracellular concentration of carbon, nitrogen, phosphorus, silicon and chlorophyll. The health of the cell is of course defined by the intracellular quota of all elements^66^, although carbon is the fundamental “currency” of life, that is, the leading element in the BFM framework.

The deterministic BFM dynamics presents a cyclic behaviour for all the interacting plankton functional types^54^. The deterministic oscillations, at the steady state, are related to the seasonally periodic behaviour of solar irradiance and environmental temperature. The former oscillates with a seasonal period between a lower value during winter ($100 ~ \mu E \cdot m^{-2}$) and a higher value during summer ($700 ~ \mu E \cdot m^{-2}$), with a superimposed day-night light cycle (see panel B in Fig. 7). Analogously, temperature (*T*(*t*)) deterministically varies over one-year period ($T_{det}(t)$) with a minimum value in winter ($8~^\circ C$) and a maximum value in summer ($28~^\circ C$) (panel B in Fig. 7).

The system dynamics is strongly influenced by the downward solar irradiance. In winter, indeed, the system is limited by light with diatoms and nanoflagellates showing very low concentrations. After the cold period, when light is no longer limiting while nutrients are, picophytoplankton (P3) dominates among the primary producers, winning the competition for nutrients (in particular nitrates) (see panels C and D in Fig. 7). Dinoflagellates are virtually extinct because of their low growth rate. The predation, according to the trophic web shown in Fig. 7, modulates in summer the biomass of the microbial loop compartment (bacteria and picophytoplankton), which shows sustained biomass during all the rest of the year. It is worth pointing out that no intrinsic oscillations would be present in the ecosystem dynamics if the seasonal forcing-induced variability were neglected.

In order to describe such a complex network dynamics the BFM is a system of 54 nonlinear ordinary differential equations (ODEs). For more specific details and the full list of equations and processes included in the BFM, Ref.^41^ and the BFM code manual^5^ are recommended. The standard formulation of light limitation used in BFM, which is based on a saturating formulation^67^, has been extended in the present work with a photoinhibition term^68^ to account for cell degradation related to high light exposure. To understand the role of photoinhibition we performed a sensitivity analysis and verified that such effects have low impact on the ecosystem dynamics.

### The stochastic version of the biogeochemical flux model

Considering the solar irradiance as a stochastic process leads to the stochastic version of the Biogeochemical Flux Model (SBFM). In this work the irradiance is considered to be influenced by a multiplicative self-correlated Gaussian noise. The choice of a multiplicative noise is simply due to the fact that the light signal vanishes during the night and then the noise intensity must necessarily vanish as well. The random fluctuating light can be formally described by the following Langevin equation

1 $$L\left( t \right) = L_{{\det }} \left( t \right)[1 + F_{L} (t)],$$

2 $$\begin{aligned} \frac{dF_{L}(t)}{dt}= & {} -\frac{F_{L}}{\tau }+\frac{\xi _L(t)}{\tau }, \quad F_{L}(0)=0, \end{aligned}$$

with $\tau$ being the correlation time and $\xi _{L}$ the white Gaussian noise with mean value and correlation function given by

3 $$\begin{aligned}<\xi _{L}(t)>=0; <\xi _{L}(t)\xi _{L}(t')>=D\delta (t-t'). \end{aligned}$$

Here *D* is the intensity of a white Gaussian noise source (expressed in *second*), and $F_{L}$ the randomly fluctuating component of the irradiance.

It is important to underline that $F_{L}$ cannot become smaller than -1 since in this case physically unacceptable values of light irradiance, that is negative values, would be reached. For this reason we imposed $[-1,1]$ as range of variation of $F_L$. The upper bound is not strictly necessary in principle, however Fig. 1c indicates that random fluctuations do not exceed the $\pm 100\%$ limit. Moreover, a symmetric range of variation ensures a Gaussian distribution around the (almost) vanishing mean value.

The stochastic process we consider in Eq. (2) corresponds to the so called Ornstein-Uhlenbeck process, which can be interpreted as a damped Brownian motion. In this case the damping parameter is characterized by the temporal scale $\tau$. The Ornstein-Uhlenbeck process presents analytical expressions for the mean value and the variance^69^, namely:

4 $$\begin{aligned}<F_{L}(t)>= & {} <F_{L}(0)>e^{-\frac{t}{\tau }}, \end{aligned}$$

5 $$\begin{aligned} \sigma _L^2= & {} \mathrm {var} \{F_{L}(t)\}=\{\mathrm {var} \{F_{L}(0)\} - \frac{D}{2\tau }\}e^{-2\frac{t}{\tau }} + \frac{D}{2\tau }, \end{aligned}$$

where $\sigma _L$ is the amplitude of the light fluctuations. Asymptotically, that is for sufficiently long times ($t \gg \tau$), the average of fluctuations vanishes with the variance tending to $\frac{D}{2\tau }$. According to the Wiener-Khinchin theorem^69^, through the stationary correlation function

6 $$\begin{aligned} <F_{L}(t)F_{L}(t')>=\frac{D}{2\tau }e^{-\frac{|t-t'|}{\tau }}, \end{aligned}$$

the corresponding spectrum (S) can be computed as

7 $$\begin{aligned} S(\nu ,\tau ,D)=\frac{D}{2\tau } \int _{-\infty }^{+\infty }e^{-\frac{|s|}{\tau }}e^{-i 2 \pi \nu s} ds=\frac{D}{1+(2\pi \nu \tau )^2}, \end{aligned}$$

with $s=t-t'$ being the lag time and $\nu$ the frequency.

It is worth noticing that the damping term in Eq. (2) is needed since a white Gaussian noise source would cause in time an unlimited increase of the light fluctuation amplitude. Such a non-physical condition is avoided within the Ornstein-Uhlenbeck framework for $\tau \ne 0$, with $\tau \rightarrow 0$ giving again a white Gaussian noise source.

### Simulations

The model equations were integrated within a time-window of nine years. In order to neglect the transient dynamics and to focus only on the oscillating steady state (which characterizes the long-time system behaviour), in the multi-panel figures only the last five years have been considered. The mathematical complexity of the model requires that we numerically integrate the equations and average the results over a set of realizations. We chose the Ito scheme to numerically solve the Fokker-Planck equations and to perform 1000 realizations for each dynamical scenario identified by a precise couple of values for the noise intensity *D* and the correlation time parameter $\tau$.

Due to the nonlinear character of the BFM, averaging over different realizations does not rule out the effects stemming from random environmental fluctuations. In a linear model, indeed, the mean value of a generic variable is not affected by stochastic perturbations. Conversely, in the presence of nonlinearities, intriguing counterintuitive changes may be observed both for the variance and the mean value of the state variables.

## Data Availability

The experimental datasets analysed during the current study are available in the following repository: BOUSSOLE mooring, Antoine and Vellucci, 2020; http://www.obs-vlfr.fr/Boussole/html/project/boussole.php. The datasets generated during the current study are available from the corresponding author on reasonable request.

## Code availability

The code of the main deterministic version of the BFM is open source and available at https://bfm-community.github.io/www.bfm-community.eu/. Details about the stochastic version of the BFM code reproducing the key results of this paper is available from the corresponding author upon reasonable request.
